# Sparking Attention: Qualitative Evaluation of General Practitioners’ Perceptions of Rising Adult Attention Deficit Hyperactivity Disorder Presentations in a Scottish Setting

**DOI:** 10.7759/cureus.97238

**Published:** 2025-11-19

**Authors:** Calum Silcock, Thomas Leung, Andrew Radley, Stefan Clos

**Affiliations:** 1 Medicine, Queen Elizabeth University Hospital, Glasgow, GBR; 2 Psychiatry, Murray Royal Hospital, Perth, GBR; 3 Public Health, Tayside Directorate of Public Health, King's Cross Hospital, Dundee, GBR; 4 Psychiatry, Taranaki District Hospital Trust, Taranaki, NZL

**Keywords:** adult adhd, attention deficit hyperactivity disorder (adhd), general practitioners, primary health care, qualitative research

## Abstract

Background

Adult presentations for attention-deficit/hyperactivity disorder (ADHD) are perceived to be rising, placing pressure on services. General practitioners (GPs) are often the first point of contact and frequently collaborate with specialty care in ongoing management. We explored GP perspectives on drivers of demand and barriers to safe, coordinated care within the National Health Service (NHS).

Methods

We aimed to investigate GP perspectives on diagnosis and management of adult ADHD. We conducted a qualitative study with analysis initially drawing on grounded theory principles (inductive coding, constant comparison, and iterative refinement of topic guides), whilst later using the framework method to provide a systematic structure for analysis. Semi-structured video interviews were completed with 10 GPs and two psychiatrists from NHS Tayside (Scotland) from February to April 2022. Participants were purposively sampled by GP age, experience, practice size, and deprivation level. Transcripts were independently coded by two or more researchers, and a consensus analytical matrix was developed to identify themes across demographics and professional groups.

Results

Participants reported increased adult presentations for suspected ADHD, often prompted by self-identification, peer influence, or media exposure. Deprivation was viewed as the demographic factor with the greatest impact on need, whereas age and sex had modest effects. GPs described limited training and reliance on rating scales to support referral, alongside long waits and fragmented communication with psychiatry. Shared-care arrangements were inconsistently operationalised: most GPs expected psychiatrists to provide follow-up monitoring, while psychiatrists expected GPs to conduct physical health monitoring. Transition from child and adolescent mental health services (CAMHS) to adult services was a recurrent vulnerability, with young adults frequently “lost to follow-up.” Patients commonly sought private assessment to bypass delays, raising concerns about variable quality and equity. GPs perceived medication benefits as largely subjective but generally positive, while expressing unease about under-monitoring and potential misuse.

Conclusions

GPs perceived rising adult ADHD demand driven by social awareness and self-identification, occurring within strained services and unclear monitoring responsibilities. Clarifying roles within shared care, strengthening CAMHS-to-adult transitions, and expanding multidisciplinary capacity may reduce waits, improve safety, and support equitable access to evidence-informed care.

## Introduction

Attention-deficit/hyperactivity disorder (ADHD) is an increasingly prevalent psychiatric diagnosis in adults, characterised by hyperactivity, impulsivity, and inattention across multiple settings [1]. Although clinicians most often diagnose ADHD in childhood, and diagnostic criteria require the presence of symptoms before age 12, symptoms frequently persist into adulthood [2]. The estimated prevalence is approximately 5% in childhood [3] and 2.5% in adulthood [2]. Over the past 20 years, diagnostic rates have increased in many countries, including the United Kingdom (UK) [4]. Prescribing rates for adult ADHD vary by country but increased in Scotland [5], Europe more broadly (from 17% to 19%), North America (from 11% to 15%), and Asia and Australia (approximately 25%) between 2001 and 2015 [6].

In most of the UK, primary and secondary care share responsibility for adult ADHD, with both pharmacological and psychological treatments available [7]. The National Institute for Health and Care Excellence (NICE) outlines primary care roles (i.e., initial recognition, referral, continuation of prescribing, and monitoring) while secondary care leads diagnosis, treatment initiation, titration, and planning [8]. Shared-care agreements delineate these responsibilities so that general practitioners (GPs) follow routine patients while secondary care manages complex cases, with joint decision-making around prescribing [9]. Some ADHD shared care agreements may not be accepted by GPs due to doubts around diagnosis, availability of treatment, inconsistent monitoring [10], and insufficient resources to manage increased workload [11]. Funding allocation and medication costs likely contribute to regional variation: some UK areas provide specialist tertiary ADHD services, whereas others place greater responsibility on primary care for ADHD management [9].

The upward trend in diagnosis and treatment raises several questions. Some analyses suggest that apparent increases reflect methodological and diagnostic heterogeneity rather than true changes in prevalence [12]. Other contributors may include greater awareness among clinicians, teachers, and parents; improved access to healthcare; administrative practices; and broader social changes [2]. Poor-quality assessment can lead to inappropriate ADHD diagnoses [11]. Resources for people questioning a diagnosis may also be unhelpful: self-report scales can overestimate prevalence by a factor of seven to ten [13], and misinformation about ADHD is common on social media [14]. Some authors link rising diagnosis rates to social spread, noting that self-reported inattention is highly susceptible to peer influence [15].

Social contagion (i.e., the spread of noncommunicable behaviours, concepts, and conditions through social influence) may help explain these patterns [16]. Under this theory, symptom levels may remain stable while the perceived meaning of and potential for acting on a condition increase. Diagnoses may then appear epidemic, even if presentations reflect newly labelled, pre-existing symptom patterns [17], facilitated by greater treatment access. Discussion of ADHD as socially determined is emotive and controversial. ADHD likely arises from interactions between predisposed traits and environment, with social context shaping whether particular traits are conceptualised as symptoms [18]. Through this lens, the broader application of the ADHD label may reflect not a growing symptom burden but a socially transmitted shift in how traits are interpreted as diagnostic of ADHD.

GPs are often the first clinicians to assess adults with ADHD symptoms and increasingly manage their care. In the context of rapid growth in diagnosis and prescribing, this study explores GP perspectives on factors influencing trends in adult ADHD care through qualitative inquiry. We aimed to examine determinants of GP decision-making in assessment, prescribing, and referral to psychiatry services for adults who may have ADHD. Specifically, we sought to understand how GPs perceive the drivers of increased ADHD presentations, barriers to care, and role expectations in shared-care pathways.

## Materials and methods

Design

We used a grounded theory-informed qualitative methodology to capture the perspectives of GPs and psychiatrists. Data were analysed with inductive coding using a constant comparative technique, building to theory generation. We conducted thematic analysis using the framework method, following Johnson et al. [19], which enabled in-depth interrogation of the data and identification of overarching themes. We selected participants from a pre-specified sampling frame and conducted one-to-one semi-structured interviews to build an inductive understanding of opinions and salient themes, while avoiding the disadvantages of focus groups (e.g., social pressures on responses). A single researcher (CS) conducted all interviews by video call using a topic guide developed from prior literature and pilot work. Data collection occurred from February to April 2022. The research team clustered GP interview characteristics based on responses; after the sixth interview, no new clusters emerged. At this time, during the consensus coding of transcripts, no further codes were found to emerge. In light of these findings, it was assessed that data saturation had been reached. The range of participant characteristics and the rigorous analytic approach yielded rich, detailed data.

Ethics

This work was performed in accordance with the Declaration of Helsinki. Ethical approval for this work was received from the University of Dundee School of Medicine and Life Science Research (SMED) Ethics Committee on January 13, 2022 (SMED REC Number 21/158). Data protection was maintained by pseudonymising data with a participant code, with data held in secure cloud storage.

Sampling

We recruited 10 GPs and two psychiatrists from the National Health Service (NHS) Tayside in east Scotland. NHS Tayside has the highest level of prescribing ADHD medications to adults of any health board in Scotland, which increased by 293.1% between 2010 and 2019 [5]. In Tayside, adults with ADHD are managed under shared care between GPs and secondary care psychiatry services (community mental health teams). Inclusion criteria were therefore qualified GPs and psychiatrists (not in training), who had worked in NHS Tayside during the period from 2010 to 2019. Exclusion criteria were doctors currently in GP or psychiatry training, GPs and psychiatrists who had started working in NHS Tayside after 2019, and doctors in other fields of medicine.

Recruitment occurred via email, with university contacts forwarding invitations to generic GP and psychiatry mailing lists. Participants were informed of the study aims and that adult ADHD prescribing was higher in Tayside than in the rest of the country [5]. CS communicated by email to discuss inclusion and consent, including agreement to be recorded, and then arranged an online interview. Informed consent was taken from all participants. Participants received no incentives. One hundred forty-two potential participants were contacted. The responding sample initially included 11 GPs and four psychiatrists, giving a response rate of 10.6%. One GP and two psychiatrists later withdrew because of time constraints (n=2) and loss to follow-up (n=1).

We categorised responding GPs by age, sex, length of professional experience, practice size, and deprivation levels. This approach guided further recruitment to fill demographic gaps. Because female GPs and newly qualified GPs (<10 years since qualifying) were underrepresented, we sought additional participants from these groups via a local primary care newsletter. This purposive sampling produced a sample of 10 GPs that was representative by GP age and practice characteristics (see Tables 1 and 2). Table 1 outlines demographic data for individual GP participants. Table 2 outlines practice size and deprivation data for practice populations; deprivation was assessed using the Scottish Index of Multiple Deprivation (SIMD) [20]. The SIMD is a composite measure of relative socioeconomic deprivation (lack of material benefits), measured by access to income, employment, education, health, access to services, crime and housing [20]. Individual GP participant data allowed responses to be contextualised by professional experience and demographic characteristics. GP practice deprivation and size data allowed for clustering of responses within the socioeconomic context in which they were produced.

**Table 1:** Characteristics of GP participants (n=10) GP - general practitioner

Characteristic	Value
Female, n (%)	3 (30%)
Male, n (%)	7 (70%)
Median age, years (range)	51 (36–61)
Median years in GP practice (range)	20.5 (1–34)
Median years’ experience in psychiatry (range)	0.5 (0.5–20.5)
Psychiatry rotation completed during GP training, n (%)	4 (40%)

**Table 2:** Individual practice characteristics (n=10) SIMD - Scottish Index of Multiple Deprivation

Characteristic	Category	Definition	n (%)
Practice population size	Small	<5,000 patients	4 (40%)
	Medium	5,000–10,000 patients	3 (30%)
	Large	>10,000 patients	3 (30%)
Deprivation (SIMD quintile)	High	SIMD 1 (most deprived)	3 (30%)
	Medium	SIMD 2–3	6 (60%)
	Low	SIMD 4–5 (least deprived)	1 (10%)

Psychiatry participants were recruited in a similar manner via email communication through university contacts. In line with the aims of the study, fewer psychiatrists were recruited than GPs in order to provide a comparative group. The demographic characteristics of the psychiatry participants are summarised in Table 3. 

**Table 3:** Characteristics of psychiatry participants (n=2)

Characteristic	Value
Female, n (%)	1 (50%)
Male, n (%)	1 (50%)
Median age, years (range)	53.5 (53–54)
Median years in psychiatry (range)	24 (20–28)

Data collection

We conducted single, one-to-one online interviews using Microsoft Teams (Microsoft Corp., Redmond, US). Interviews averaged 37 minutes (range: 30 to 50 minutes) and were conducted from February to April 2022. Interviews were recorded and transcribed verbatim, with contextual field notes taken during the process; notes were destroyed after transcription.

Authors CS and SC developed the initial topic guide, informed by a pilot interview. Using a constant-comparative technique, we compared each case with prior cases and the guide. Topics raised in early interviews that were not initially included were added to subsequent interviews, yielding the final topic guide (Table 4). Although both CS and SC initially planned to conduct interviews, we determined that SC’s role as a potential clinical colleague might influence responses through social desirability bias. It was felt that practicing participants may be more open when discussing topics with an early-career researcher without a professional relationship with them. Therefore, CS, a male Bachelor of Medicine, Bachelor of Surgery (MBChB) student, conducted all interviews. This reflexive consideration of researcher positionality reduced the risk of biasing participant responses [21]. Furthermore, it was considered how prior knowledge, experiences, and assumptions of each of the research team members could influence data interpretation; regular discussion encouraged reflexive questioning of emerging codes and themes to mitigate potential biases and ensure that findings remained grounded in participants’ accounts.

**Table 4:** Final summary topic guide ADHD - attention-deficit/hyperactivity disorder; GP - general practitioner

Heading	Issues covered
Adult ADHD treatments	Challenges in treating; aims of prescribing; role of the GP; growth in prescribing
GP factors in referral and prescribing	GP experience; training in ADHD; use of screening tools
Patient and external factors in referral and prescribing	Patient expectations; demographics of typical patient; influence of press/media/industry/colleagues; guidelines; formulary
Practical prescribing	Co-prescribing of other medicines; drug–drug interactions; monitoring of prescribed ADHD medicines

Analysis

Adopting a pragmatic epistemological stance, analysis during data collection drew on principles from grounded theory: inductive coding and constant comparative technique allowed concepts to emerge from the data, which informed iterative refinement of the interview topic guide. Subsequently, the framework method was used to systematically organise emergent codes and support transparent, structured analysis. During initial coding, each researcher independently assigned short descriptive open codes to text segments. At least two team members coded every transcript. The team then discussed each transcript and compared codes to reach a consensus interpretation, organised in a codebook (Table 5).

**Table 5:** Codebook used in framework analysis of interviews ADHD - attention-deficit/hyperactivity disorder; BP - blood pressure; CAMHS - child and adolescent mental health services; DSM - Diagnostic and Statistical Manual of Mental Disorders; Dx - diagnosis; GP - general practitioner; HR - heart rate; NHS - National Health Service; Rx - prescription/medication; SIMD - Scottish Index of Multiple Deprivation; Tx - treatment

Category	Subcategory	Codes (representative concepts)
Disease factors	Diagnosis	Diagnostic clouding; diagnostic threshold; diagnosis without medication (Dx without Rx); expanding diagnosis; retrospective diagnosis; self-diagnosis; specificity of diagnosis; under-diagnosis; validity of diagnosis; validity of ADHD diagnosis; validity of psychiatric diagnoses (general)
	Natural history	Course of illness/ natural history/ recognition (perceived evolution over time)
	Neurodiversity and non-pharmacologic care	Neurodiversity; non-pharmacological treatment (Tx); significance of neuroimaging
	Symptoms	Functional impairment; subjective symptom burden
GP factors	Training and confidence	Lack of training; low confidence in diagnosing ADHD
	Guidance and systems	Impact of guidelines; formulary constraints; computer-supported prescribing
	Prescribing posture (vs. colleagues)	Prescribes less than colleagues; prescribes more than colleagues; same as colleagues; unaware of relative level
	Referral tools and threshold	Reluctance to refer; awareness of short screening tools; not aware of short screening tools
Medication factors	Benefits and risks	Perceived benefits (focus, control, mood/relationships); adverse effects (anxiety); adverse effects (cardiac); financial impact
	Monitoring and safety	Physical monitoring expectations; dispensing controls; polypharmacy concerns; subjective assessment of response
	Misuse	Misuse/overuse; diversion / performance-enhancing use (concern)
Patient factors	Age and sex	Child vs adult (different presentations, pathways, and thresholds); age (modest impact on suspicion/referral); sex—men more than women (historical); sex—approximately equal (contemporary view)
	Context and comorbidity	Psychiatric comorbidity—none reported (in some presentations)
	Motivations and setting	Secondary gain (e.g., accommodations, work protections); medication-seeking; students (university context)
	Preferences	Non-pharmacological strategies sought/valued
Psychiatry factors	Perspectives and resources	Diverging opinions (GP vs psychiatry); psychiatric input influences GP referral
	Role of secondary care	Transition (CAMHS → adult); under-resourced services; referral not altered despite resource strain (some GPs)
	Shared care	Communication (clarity of roles; feedback to primary care)
Socio-cultural factors	Cultural and media influence	American influence; media; internet/social media; medical press
	Awareness and education	Public awareness; parental understanding; stigma (decreasing)
	Systemic and structural	Role of NHS; private-care pathways; pharmaceutical influence; no perceived pharmaceutical influence; deprivation (area-level)
Psychosocial factors	Subjective suffering	Psychosocial contributors to perceived impairment
Trends in ADHD	Presentation and labeling	Increased diagnosis/presentations; neurodiversity (as framing)

After consensus coding for a transcript, we imported the agreed codes into NVivo 12.6 [22] to support further analysis, including the construction of the analytical framework and the analytical matrix. We applied the framework method of analysis to enable collaboration among researchers from different backgrounds and to facilitate comparisons across demographic groups. We created the analytical matrix in NVivo (QSR International, Melbourne, Australia) and Microsoft Excel (Microsoft Inc., Redmond, US) to visually represent the data. The research team reviewed the matrix to identify patterns and themes across the dataset and compared responses of GPs and psychiatrists. Consensus codes were grouped into categories, which aided in identifying emerging themes. For example, text relating to blood pressure and heart rate was coded initially as “cardiovascular,” which was categorised under the subtheme of “physical monitoring.” This was felt to be related to, yet distinct from, “dispensing,” and both were grouped under the same major theme of “shared care.” Through iterative discussion, related codes were grouped into categories, which were then refined and clustered into overarching themes that were checked against the original transcripts to ensure they accurately reflected participants’ perspectives. Where opinions were shared across several participants, we recorded descriptive frequencies as a proportion of the whole, small, purposive sample.

We enhanced accuracy and reliability by seeking disconfirming cases and clustering GP opinions. For example, we observed that GPs who did not mention the use of private care services were more likely to work in practices serving deprived areas. We also assessed inter-coder consistency; many text segments received similar codes from multiple researchers.

## Results

Analysis of interview data indicates that GPs perceive increased demand for adult ADHD diagnosis and referral within a health system that cannot meet current demand. Patients often take proactive steps to obtain an ADHD diagnosis, further pressuring already busy services. GP decision-making is necessarily influenced by personal experience (individual, training, and system-level), leading to idiosyncratic referral patterns.

**Figure 1:**
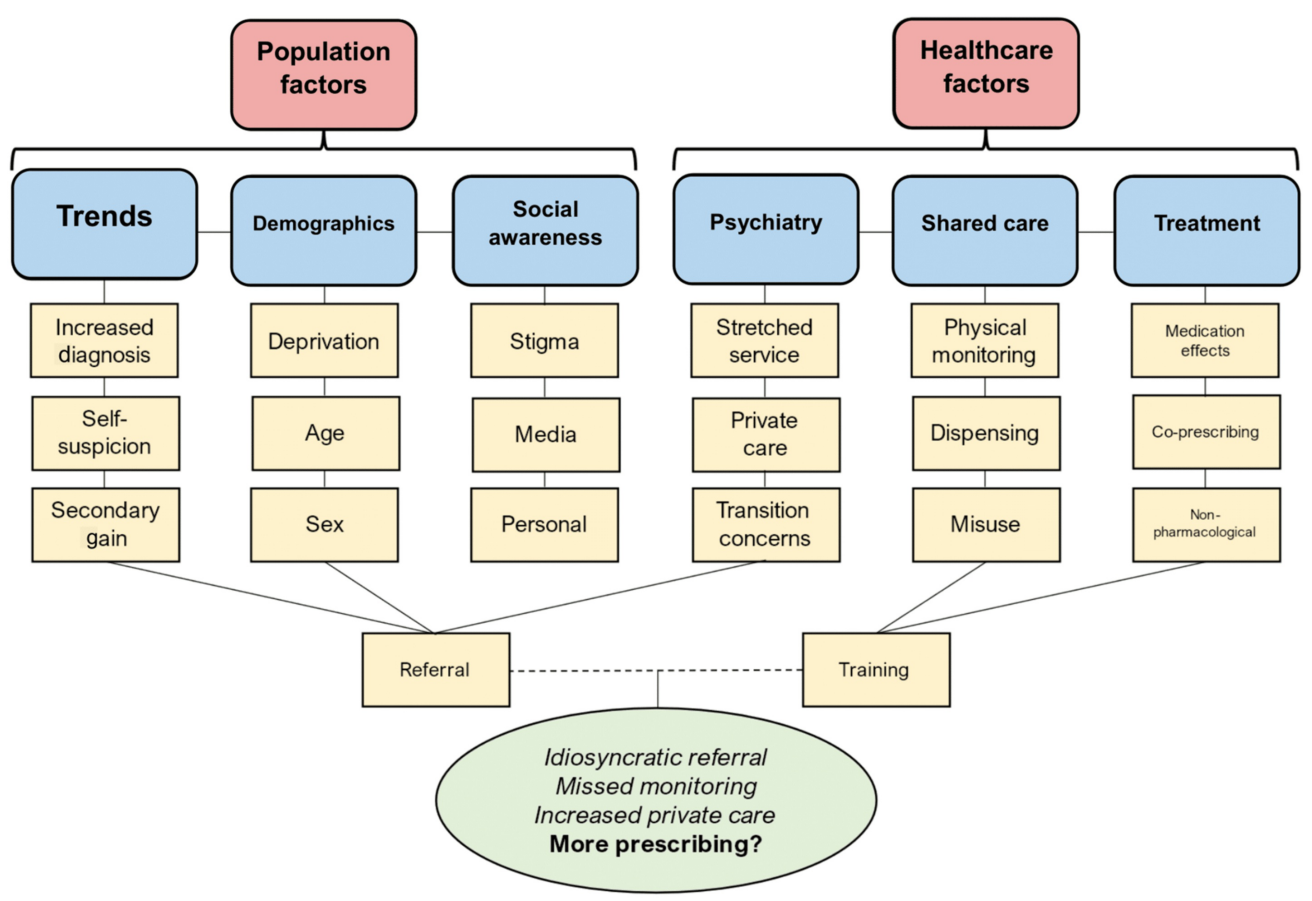
Results of framework analysis Threads (red), themes (blue), and sub-themes (yellow) developed during the process. Synthesis of data is seen in the oval (green). Lines indicate related or derivative concepts.

We organised the analysis into two broad threads, population factors and healthcare factors, to reflect the interwoven influences on patient-GP interactions. Within population factors, GPs discussed trends in ADHD, patient demographics, and societal awareness. Within healthcare factors, GPs discussed psychiatry services, shared care, and treatment. Each theme included several interrelated subthemes, and some subthemes spanned multiple themes, contributing to a synthesis of the dataset (Figure 1).

Grouping

**Figure 2:**
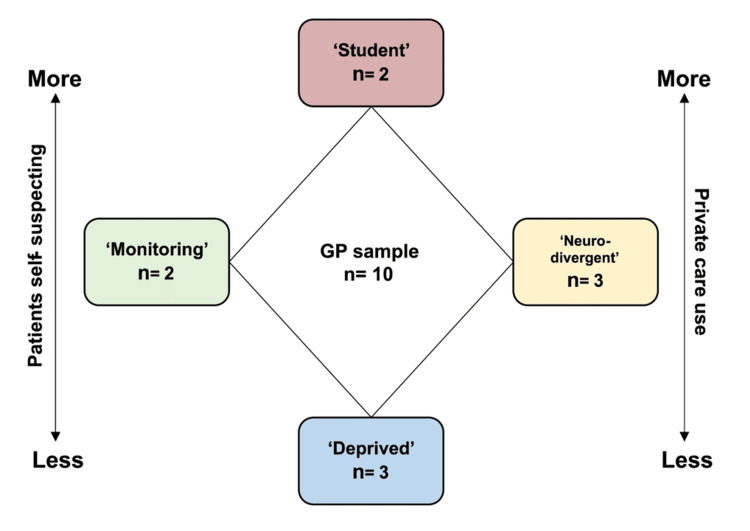
GP clusters and associations These clusters were produced intuitively and did not appear to correlate with individual GP characteristics. Relationships reflect participant framing: GPs in the “student” group frequently tended to emphasise their patients self-suspecting ADHD and private care use, the “monitoring” and “neurodivergent” groups addressed these topics to a moderate extent,  whilst those labelled as “deprived” mentioned these topics very little or not at all. GP - general practitioner

We empirically clustered GP participants into four groups with broadly comparable experiences and opinions based on interview content (Figure 2). The “student” group self-identified as working in university areas; the “neurodivergent” group reported personal experiences with ADHD or used that term; the “deprived” group self-identified as working in deprived practices; and the “monitoring” group emphasised physical monitoring in this patient population. This clustering emerged during the disconfirmation process and appeared to align with the use of private care and with subjective estimates of the proportion of patients who suspected they had ADHD. The ability to cluster GPs by similar opinions and the absence of new clusters after the sixth interview indicated the full range of GP opinion had likely been captured. Participants’ opinions and experiences following the sixth interview each aligned with already defined clusters, indicating data saturation by empirical clustering. Saturation was confirmed by analysis of later transcripts frequently using codes and identifying themes already laid out in earlier interviews, suggesting a thorough assessment of perspectives in the area.

Increased diagnosis

All GPs perceived an increase in the number of adults with ADHD compared with earlier periods. Although diagnoses had been rare in the past, GPs emphasised that they are still not seeing large numbers overall. More experienced GPs described this rise relative to the start of their careers, when adult ADHD was emerging in practice. More recently qualified GPs expressed concern that training and resources may not fully equip them to address patient queries.

“Certainly we have seen an awful lot more people… an awful lot more children with ADHD over the years. It used to be a very rare diagnosis. Now we have quite a number, but diagnosing it in adults is something that I’ve maybe only seen much more recently, maybe in the past ten-fifteen years.” (GP3)

Some GPs perceived that populations from some countries, particularly in North America, are more likely to receive ADHD medications. Five of 10 GPs suggested that cultural and social influences from these countries may contribute to increased patient awareness in Scotland, particularly among students and through media exposure.

“I mean, this big spike. We saw it in the US like 10 years ago, right? So it’s not a massive surprise. It’s just you might almost say- I don’t know? Has the UK ‘caught’ ADHD?” (GP1)

Social influence

GPs reported that decreased stigma, more positive media representation, and greater personal familiarity with ADHD contribute to diagnostic trends. Increased awareness of neurodiversity was viewed as a key driver of adult presentations. Participants described society as more accepting of mental health issues than in the past, enabling more open discussion of symptoms, diagnoses, and medications and reducing stigma. These shifts were seen as legitimizing patient experiences and providing an explanatory label that can increase adult diagnosis.

“There’s a lot more talk of mental health awareness and how common it is and how people should present and go and see about it. And I suppose there is a lot more of that in general. Yes, I think the stigma is getting much less.” (GP6)

GPs attributed increased awareness of ADHD to media exposure, including social media, websites, newspapers, television, and radio. “High profile discussion” (GP3) was perceived as especially influential, linked to pop culture and internet culture. 

“You know, the more people realise it, the more they talk to their friends, the more that it becomes publicised, especially when it’s on the telly on mainstream media, that people start thinking, ‘Oh, I wonder about me.’” (GP7)

Media were seen to raise awareness among both patients and clinicians and to decrease stigma. GPs acknowledged that popular media may shape background views but reported relying on professional sources to guide management, while remaining vigilant about misinformation.

“You can definitely tell if there has been a news article, or something has been publicly said- you then get a trickle of people suddenly after that coming along, so I think the media definitely has a role.” (GP6)

GP participants also observed that knowing someone who takes ADHD medication often precedes a patient’s own pursuit of diagnosis and treatment. They described this socially influenced pattern as particularly common among students, especially those with North American peers. A culturally transmitted view that medication offers solutions to challenging situations may prompt patients to seek pharmacological treatment for attentional symptoms when they might not have done so previously.

“…the more you prescribe, the more people will know of somebody who takes medication. So that kind of barrier to starting treatment is reduced.” (GP9)

“We had a lot of American students who would come over and they were already diagnosed with ADHD, and would come over requesting their medication. And then inevitably because they mixed with the ‘native’ students, the British students, British students would come and asked to get referred for assessment as well.” (GP7)

Self-diagnosis

Respondents reported that adults who receive an ADHD diagnosis often first suspect the condition themselves and bring concerns to their GP, rather than presenting with unexplained symptoms. GPs rarely identify ADHD unprompted. Participants noted that patients may complete online diagnostic questionnaires (six of 10): “there’s a lot of these questionnaires that you can administer yourself online now” (GP6), or seek private diagnosis (eight of 10): “I’ve got loads of people who get privately prescribed stimulants” (GP1), to expedite access to care. GPs viewed both routes as socially influenced. University-educated groups, including students, were described as more likely to suggest they have ADHD.

“I’ve not had that many people, adult-wise who kind of come to me and I’ve thought, ‘oh, you’ve got ADHD,’ and referred them on. I’ve had maybe one or two saying, ‘I think I’ve got it’ and ‘will you refer me?’” (GP4)

“…some of them are holding down managerial jobs and all sorts of things as well. So not always what you would typically think of someone that struggles either. Sometimes it’s been quite surprising when they’ve said, ‘actually, I find this really difficult.’” (GP6)

Some interviewees framed this pattern as patient empowerment, whereas others viewed ADHD as “essentially a self-diagnosable condition” (GP1), questioning the validity of an expanding diagnosis. Most GPs were unlikely to refute a potential diagnosis, particularly when patients had gathered evidence or were distressed. They perceived that some patients seek an ADHD diagnosis not solely for symptom relief but for perceived advantages in personal or professional contexts (secondary gain).

Secondary gain was rarely suspected to involve drug-seeking. More commonly, patients sought a diagnosis for perceived job protection, access to financial benefits, educational accommodations (e.g., extra time in examinations), or an “objective diagnosis” (GP1) to explain subjective experiences. Some GPs felt patients attributed difficulties to ADHD: “‘I’ve got this condition and I can’t help it. It’s nothing to do with me.’” (GP3)

“Obviously because it effectively counts as a disability, they have certain protections and things through the workplace, so there are people who do want diagnosis just to have a diagnosis rather than necessarily going on medication or anything like that.” (GP6)

Training

Most GPs reported feeling insufficiently equipped to support or refute patients’ diagnostic queries. They described limited confidence due to inadequate training, unfamiliarity with diagnostic criteria, and reluctance to dismiss patients’ concerns. As a result, most preferred seeking a secondary care opinion rather than making diagnostic decisions themselves.

“I don’t feel I’m in a position to remove a diagnosis or say definitively not.” (GP5)

Seven of 10 GPs identified inadequate training in adult ADHD as affecting practice; they “don’t feel confident enough” (GP3), particularly regarding diagnosis. Consequences included more referrals to psychiatry for borderline cases and limited awareness of drug monitoring and co-medication issues. Four of 10 GPs had specifically sought learning materials on adult ADHD, and a further two of 10 reported they “had probably read a few bits and pieces that have come out on it” (GP6) in medical journals. Others cited time pressure and relatively low patient numbers as barriers to further learning.

“I think ADHD has been possibly a topic that was not well studied through our undergraduate training, and probably only just touched on in our postgraduate training. So I just generally think that it’s not a topic that’s been explored fully.” (GP8)

“…although I go to a lot of additional [psychiatry] meetings on top of the usual stuff, I don’t remember seeing something specifically for ADHD.” (GP3)

Demographics

Participants reported that patient demographics influenced both initial help-seeking and referral to psychiatry. In order of perceived impact, the key factors were: (1) level of deprivation, (2) age, and (3) sex.

Deprivation

In this sample, GPs perceived that well-educated people and students were more likely to request a referral for ADHD, with social contagion compounding effects in these groups. By contrast, the patients in whom GPs most often suspected ADHD were those from deprived areas with chaotic histories and difficult family backgrounds. Although GPs often felt that ADHD might be an appropriate diagnosis- “usually significantly deprived, long history of difficulties” (GP1)- patients in these circumstances tended to prioritise other issues such as “substance misuse or unemployment.” (GP4)

“I have several patients who I strongly suspect their chaotic lives are possibly a result of ADHD, but they don’t have a diagnosis. They’ve just got massive mental health problems.” (GP7)

This sample of GPs perceived that deprived patients were less likely to receive an ADHD diagnosis because of psychiatric comorbidity, complex social histories, competing priorities, higher stigma, and time pressures in primary care. These patients were also considered less likely to use private services.

Age

GPs reported that patient age had a modest influence on their likelihood to suspect ADHD and to refer to psychiatry. They most often expected adult presentations in “the late teens and 20s to 30s” (GP2), with decreasing likelihood beyond middle age: “The older the adult, as I say, again, when I have fifty-sixty-seventy-eighty-year-olds, I won’t really be thinking about ADHD.” (GP4)

GPs did not view age as an independent determinant of presentation. Rather, age interacted with other demographic and social factors. For example, they attributed increased diagnoses in the early 20s to challenges associated with tertiary education. Patients in their 30s were more likely to have children with ADHD, prompting self-reflection and help-seeking. Despite these expectations, age did not determine referral decisions:

“… there’s an awful lot of things in medicine where they say, “up to this age… over that age.” But you know, if someone’s got symptoms, they’ve got symptoms regardless of what their age is.” (GP3)

Sex

Overall, GPs reported they suspected ADHD in men and women with similar frequency. Some noted that historical impressions may still yield slightly higher diagnosis rates in men, but they described the gap as narrowing. Consequently, sex had a weak impact on referral.

“I mean it obviously used to be more blokes, but now… it’s now about 50:50 or so… maybe we’re still at the 60:40 point blokes, maybe, but it’s getting close to 50:50.” (GP1)

“…you may be talking about a risk or an epidemiological factor, but your immediate action point is you need to sort out his [symptoms]. And it’s a little bit like that with ADHD, whether they’re male or female, well, it can actually affect both sexes, so you need to act accordingly.” (GP8)

A minority of GPs (three of 10) discussed sex differences in adult presentation, suggesting that classical descriptions may not reflect women’s experiences. These GPs also discussed personal experiences with ADHD, neurodiversity, or both. Two had been qualified for less than two years, suggesting recent training may highlight these issues.

Healthcare factors

Healthcare factors encompassed private and NHS psychiatry, shared care, and medication. All GPs reported that local General Adult Psychiatry services were under pressure, citing long waiting times (eight of 10), low numbers of psychiatrists (three of 10), poor continuity of care (three of 10), and high use of locum staff (two of 10). They emphasised that these systemic issues affected patients with “really severe psychiatric problems” (GP1) and “absolute mental health crises” (GP7), as well as affecting those with suspected ADHD. Delays were particularly consequential when patients presented during periods of life change (e.g., starting a new job or educational institution):

“… they’re going to be waiting for a long time, the idealist 12 to 14 week waiting time list is just non-existent within psychiatry. In our cluster, you’ll probably be waiting in the region of somewhere of four to six months minimum to see somebody once, that you’ll never meet again because they’ll have changed by then.” (GP2)

Private care

Service pressures influenced both GP and patient behaviour, affecting referral likelihood, referral pathways, and the advice provided. Patients were reportedly more likely to seek private care or disengage. GP participants reported that long waits led “patients [to] lose faith” (GP8), prompting patients to seek alternative routes, such as private or occupational health services. Eight of 10 GPs had patients who pursued private diagnosis or management. This response often followed prolonged waits, leaving some patients “desperate” (GP9) and in “misery” (GP1). Despite this, private services were not necessarily felt to benefit patients:

“We have seen quite a few [patients] that have gone the private route, which we have actually actively discouraged because we don't know how reliable the diagnosis is in the private sector, and then if they transfer across the NHS for further care, will it all just get thrown out? And yeah, it's a whole mess, it really is.” (GP6)

Both GPs who did not mention private care worked in practices in Scottish Index of Multiple Deprivation Quintile 1 (the highest deprivation quintile). Two GPs criticised private services, citing lower standards and pay-to-access models that fostered a “cynical” (GP1) view. These two GPs self-identified as working in student areas and expressed reluctance to refer for ADHD, which may have contributed to their perspectives.

Transition concern

Participants identified limited service provision as particularly problematic for patients transitioning from child and adolescent mental health services (CAMHS) to adult psychiatry, where there was “this sort of hole between 16 and 18” (GP3). GPs expected that when young adults who were closely followed by CAMHS “cross that threshold from the paediatric service” (GP2), their age, education, or employment status would mean “they will be discharged” (GP2). These patients were expected to default back to primary care and were often “lost to follow-up” (GP3) from psychiatry services.

“I'm not convinced that the adult psychiatric service … is particularly great at picking that up or indeed at all. They just kinda say, “Yeah, you're an adult now, go to your GP, cheerio”, unless there happens to be a concurrent other mental health issue that may play into it.” (GP4)

GPs who expressed concern about transition consistently highlighted unclear communication. They perceived gaps not only between psychiatry and general practice but also within psychiatry services. As a result, GPs were uncertain about the plan for ADHD care after CAMHS discharge, while “sometimes the adult psychiatric service doesn’t seem to think they have a role for those people.” (GP3) Responsibility for ongoing ADHD management was unclear, and GPs worried this ambiguity would harm mental and social wellbeing.

“One area where I think we’re not 100% clear on is how the transition between child and adult services works for them as well. It doesn’t always seem to be quite clear from correspondence what’s happening.” (GP6)

Because of the combination of life stage, lack of a clear transition pathway, and symptom impact, this group of GPs perceived early adulthood as the period of highest risk for disengagement from services and cessation of ADHD treatment. While an older view “assumed that it was mainly a childhood illness, and it kind of burns itself out” (GP9), participants expressed concern that disengagement represents a missed opportunity to prevent later health, social, and employment problems.

“I’m concerned that some of these people further down the line are going to have problems at that stage, and then realise that maybe life would have been different if I had stayed on medication.” (GP3)

Shared care

Monitoring knowledge varied among GPs. Four of 10 GPs named physical parameters to monitor-blood pressure (BP), heart rate (HR), and/or weight-whereas two reported they did not know which physical parameters to monitor. Most GPs (seven of 10) expected psychiatry to provide follow-up monitoring. Although they might record BP for other reasons, most would not measure it specifically for patients prescribed ADHD medication. In this context, GPs perceived their role as primarily prescription continuation for a group “not within our area of expertise at all” (GP9).

“Then it’s over to us as a letter saying, ‘will you take on prescribing for this patient?’ Yes, we will, but then they’ll presumably continue to get follow up with the psychiatrist anyway. … So we’re just dishing out a prescription essentially.” (GP4)

“And really what we’re doing is just issuing the repeat prescriptions that patients will require. But we need to know that they are engaged with psychiatry follow up to allow us to do that.” (GP8)

Only two GPs reported specifically monitoring physical parameters (BP, HR) in adults receiving stimulant medications. Both of these GPs had over double the mean reported years of experience (11.8 years).

In contrast to physical monitoring, responses indicated GPs consistently monitor dispensing at the practice level using electronic repeat-prescribing systems. They were aware of the controlled status of stimulant medications and limits on prescribing frequency (e.g., not more than once per month). GPs described contacting patients who appeared to request medication too frequently or who missed psychiatry follow-up; they were unwilling to prescribe when patients did not engage with services.

“… if I see a patient coming along and they failed to attend the last two psychiatry appointments I’m then thinking, ‘Do I really want to prescribe this drug?’ And in actual fact we often don’t, and say ‘Right, I need to speak to this patient first and re-engage with them.’” (GP8)

Some GPs expressed concern about misuse, including overuse, recreational use, diversion, and “performance-enhancing” (GP2) use. Contributing factors included prior training on drug-seeking behaviour, nonattendance at follow-up, “its street value” (GP2), and a general sense of “being a bit suspicious and a bit cautious about the treatments and about the condition.” (GP4)

“I think the biggest thing we worry about is of it being misused or overused. So although if once something is at that steady level and it’s been titrated up by the specialist, when it goes on to repeat prescribing, you can’t keep getting indefinitely.” (GP3)

Medication effects

GPs in this work perceived the effects of ADHD medication as largely subjective but mostly beneficial, while noting limited familiarity with these drugs because ADHD remains relatively uncommon within typical caseloads. Reported benefits included increased focus, reduced impulsivity, a greater sense of control, improved relationships, and mood effects, although GPs found these difficult to quantify. Some suggested that stimulant medications could produce increased focus and similar effects in people without ADHD as well.

“We’ve got lots of patients who, you know, they’re seeing a psychiatrist and say, yeah, I just occasionally take the methylphenidate when I need it- in roughly the same way that you might take an energy drink. …so, you know, is it basically a slightly powered up Red Bull? I do not know.” (GP1)

GPs in our analysis group most commonly identified potential adverse effects of stimulant medications involving cardiovascular effects, anxiety, weight loss, and sleep disturbance, though many did not name specific effects. They reported greater certainty regarding adverse effects in children than in adults, the group in whom some GPs “don't know if they need blood pressure, ECG, or bloods.” (GP4)

Most GPs were not strongly concerned about co-prescribing with ADHD medications, observing that many recipients are relatively young and take few other drugs. Several relied on prescribing software to flag potential drug-drug interactions. Concerns about polypharmacy were more common among older, more experienced GPs, especially for patients with substantial existing medication burden.

“…if they’re on long-term meds like that and then there’s an SSRI or something there's likely to be enhanced cardiac issues we should be mindful about.” (GP5)

Some GPs perceived an over-reliance on medication in ADHD management and expressed a preference for broader access to non-pharmacological options, including psychological support and improved patient education. They also expected symptoms to improve with changes in life circumstances and health behaviours (e.g., less screen time, diet, exercise, and time in nature), which they believed could lessen demand on psychiatry services.

“I think there’s a probably an undervaluing of all the environmental factors in the management and planning, and maybe an over-reliance on medication. An underappreciation of environmental factors that contribute to the pathophysiology, the aetiology, and then also for management.” (GP10)

Referral to psychiatry

All interviewed GPs (n=10) acknowledged that psychiatry services are “under the cosh” (GP4), but they varied in how this affected referral behaviour. Four GPs stated that service pressures did not change their likelihood to refer, which they based on functional impairment. GPs with personal experience of ADHD reported suspecting the diagnosis more frequently. Some described strategies to mitigate long waits, such as submitting more detailed referrals, seeking formal and informal “advice requests” from psychiatrists, and using screening tools. Others (four of 10) suggested ADHD remains underdiagnosed.

Referral for a new adult presentation was less likely among GPs working in areas with large student populations. Feedback from psychiatry, high referral volumes, and long waiting times shaped this stance. Although they expressed it differently, these GPs shared a view that many students seeking diagnosis and pharmacological treatment might be better served initially by non-pharmacological approaches. The Diagnostic and Statistical Manual of Mental Disorders (DSM) was referenced in the discussion of symptom checklists.

“Well I don’t so it’s easy. I literally don’t care. You can turn up with every single symptom of ADHD straight out of the DSM and I’m not going to start Ritalin. So it doesn’t matter.” (GP1)

“…with the patient, I would usually say: ‘I would agree with you. You do have a lot of the features of ADHD, but let’s focus on what we can do about it and improve the symptoms rather than focus on just getting a diagnosis.’” (GP10)

Comparison with psychiatry participants

The psychiatrist comparison group (n=2) provided a complementary perspective and highlighted areas of alignment and divergence. Psychiatrists, through illustrative (if not definitive) comparison, corroborated GP accounts regarding diagnostic trends, changing societal attitudes, service strain, and a preference for non-pharmacological treatment in adults (noting off-label contexts). They reported overall increases in presentations, often from patients who had self-identified after consulting online resources.

“They’ve grasped on ADHD, and they’ve gone off on the internet and self-diagnosed it and they can sometimes come in with a very clear agenda for getting medication.” (PS1)

Patients did not only seek a diagnosis of ADHD for medicines, mirroring the concept of secondary gain seen in GP participants. Psychiatrists discussed views that medications are a single tool within available for treatment, and not appropriate for all patients. They emphasised the practical implications of ADHD as the main driver for considering prescribing medication:

“The main challenge is that if you arrive at the diagnosis with the patient, it’s about explaining that [ADHD] does not necessarily mean that they get a pill and everything is fixed, or indeed that they require a pill… it’s all to do with their functional impairment” (PS1)

Psychiatrists also noted that adult ADHD had not featured in their formal training, creating management challenges; in response to rising demand, they pursued educational materials and liaised with CAMHS colleagues. They agreed that resource constraints and reliance on locum psychiatrists reduced continuity of care. Although they favoured psychological interventions, they cited limited availability.

“So you know, best practice, go with psychological input and use medicine if necessary. In reality, I don’t think there’s anyone who’s got an awful lot of psychological expertise as to what you do with adults [with ADHD].” (PS2)

Diverging opinions in the psychiatry group

Some differences emerged in the small comparative group. Psychiatrists expected to see more adult women than men, and expected GPs to conduct physical monitoring for adults taking ADHD medications. One psychiatrist suggested these differences may reflect psychiatrists’ exposure to patients with severe comorbidity, which can obscure ADHD in women initially. Divergent expectations for physical monitoring may also reflect the communication gaps reported by GPs.

“The stereotype is the ADHD boy who’s bouncing around, can’t sit in his seat, and can’t shut up, and just creates trouble. And the ADHD girl is sitting quietly in her seat, staring out the window, daydreaming, not taking anything in… one is really obvious and easy to spot… the other is very much more likely to slip under the radar but yet still continue to present with symptoms of poor concentration, poor planning as an adult… having never been picked up as a child” (PS2)

Both psychiatrists expected physical health monitoring to be performed in primary care. They understood the local shared-care agreement to specify this and considered it best practice when prescribing is undertaken on another clinician’s recommendation. Resource limitations again influenced implementation, resulting in both psychiatrists interviewed expressing a view that they would be unable to perform physical health monitoring for ADHD medicines even if it were within their remit.

“I know I’m supposed to do blood pressure, but I don’t because my view is that the GP’s are the prescribers and I’m recommending it and therefore it’s up to the prescriber” (PS1)

“I’ve worked in community mental health teams where there isn’t even a sphygmomanometer… and we also don’t have scales, so we’re supposed to monitor weight and we’re supposed to monitor blood pressure, but we don’t have the equipment to do it.” (PS1)

“I must admit I was not so hot on the physical monitoring. I would monitor in terms of asking about side effects, but I wasn’t measuring weight for example, in adults… or heart rate for example, or blood pressure, as I know some people would.” (PS2)

## Discussion

Summary

We interviewed 10 GPs and two psychiatrists. All perceived an increase in adults seeking diagnosis and treatment for ADHD. This analysis identifies several problems in the care of adults with ADHD. Pressures on psychiatry services create long waits for secondary assessment; in response, GPs use screening tools and patients pursue private care. Most GPs reported limited training, lowering the threshold for referral to psychiatry. Communication gaps and inadequate transition planning from adolescent to adult services led GPs to believe that some patients are being lost to follow-up, with care defaulting to primary care. Finally, patients receiving ADHD medication appear not to be routinely monitored for physical health, with GPs and psychiatrists each assuming the other is responsible.

Mechanisms

Our data support social contagion as an appropriate lens with which to view increased ADHD assessment and treatment seeking. Social contagion as an explanatory framework may also be argued to contribute to variability in diagnosis rates, as individuals embedded in networks where ADHD is frequently discussed are more likely to seek assessment. Although rising diagnosis may reflect recognition of pre-existing traits as symptoms [17], socially driven help-seeking may increase treatment of milder cases while hard-to-reach populations remain underserved. Our analysis, therefore, presents GP opinion as favouring social contagion over alternative explanations (such as the emergence of unmet service needs) for observed trends in adult ADHD.

Lack of training in primary care emerged as a second key factor. Many GPs reported little or no relevant training, which may lower the threshold for referral to secondary care. The NICE guidance does not specifically recommend the use of rating scales at the point of identification and referral [8]; nonetheless, our findings indicate that patients and referring GPs often use rating scales to strengthen referrals. Results from clinician-administered tests are notably inconsistent [23], a problem likely exacerbated when time-pressured GPs have limited formal training. The 2017 Royal College of Psychiatrists (RCPsych) in Scotland guideline may have inadvertently increased referrals by recommending screening tools such as the poorly discriminating Adult ADHD Self-Report Scale [24], a recommendation removed in the 2023 RCPsych guideline [25]. These findings highlight the need for clear guidance and training on when and how to refer adults with suspected ADHD.

Comparison with other literature

Prior work collated views on adult ADHD care across professional groups in a conference setting [9]; our study adds depth by focusing on GPs assessing undifferentiated patients in a period of high service demand in a Scottish health board. This work updates perspectives of GPs acting in the “gatekeeper” role of ADHD service provision [26] and shows that GPs who lack the expertise to refute a diagnosis tend to follow limited training and refer to secondary care, potentially increasing demand. Our work mirrors analogous qualitative work in this field, emphasising the challenges faced by adolescents with ADHD transitioning from CAMHS to adult psychiatry [27] and echoes concerns about adolescents transitioning to adult services aligning with descriptions of a “twilight zone” in late adolescence, with reduced service input and increased risk of criminality [28]. Our finding that patients may not be monitored is consistent with earlier reports that a lack of monitoring contributes to GP refusal of shared care [10]. We extend this by suggesting that, in some cases, shared care may be accepted in principle, but physical monitoring is still not performed, exposing patients to increased cardiovascular and broader psychiatric risk [7].

Implications and looking ahead

Future work should capture adolescents’ experiences in transition, quantify monitoring practices, and report outcomes, including safety data. Quantifying monitoring practices for adults prescribed medications, including stimulants for ADHD, would corroborate the finding from our sample that this is often missed. Furthermore, longitudinal work could further assess outcomes over the life course in patients who are only first seen by ADHD services in adulthood. Clearer communications could be achieved through stakeholder meetings to agree on locally appropriate guidance.

There is a clear shortfall in services for adults seeking treatment, with universally long waiting times in this study. Improvements could include increased training of psychiatrists, providing GPs and patients with educational materials, or refining the definition of a “trained professional” in ADHD care to include appropriately trained multidisciplinary team members, given medical staff shortages. Our finding that patients often seek diagnosis for reasons beyond symptom relief underscores the importance of care pathways in which medication is not the default. Social and online discussion of ADHD appears temporally linked to rising diagnostic demand, and not all individuals will benefit from a diagnosis. Providing patients with educational resources to share evidence-informed strategies (e.g., exercise, sleep hygiene, and diet) may aid those with mild symptoms and reduce demand on psychiatry services.

Other medical specialties have long demonstrated efficient approaches to creating condition-specific registers via data linkage. In diabetes care, this strategy has supported population-based monitoring and coordinated care between primary and secondary services on a Scotland-wide platform [29]. For ADHD, the challenge is to avoid creating a silo in which referrals accumulate on waiting lists; instead, collaboration between primary and secondary care should drive solutions. This represents an opportunity for NHS-academic collaboration to co-create and rigorously evaluate interventions and alternative care models across NHS Trusts, as suggested by Guthrie and colleagues [30].

Finally, this case study from the UK NHS raises questions about ownership and accountability for clinical practice guidelines, given limited resources for guideline development and review. A potential way forward is the adoption of a framework for rapidly updating relevant guidance across organisations to ensure that recommendations respond to emerging evidence, including at individual practice, local, and regional levels.

Strengths and limitations

This study has several strengths. We used a purposive sample stratified by GP age, experience, practice size, and deprivation level, improving generalisability. Tayside, where the study was conducted, broadly reflects Scotland’s population with an approximately 50:50 urban-rural split and varied deprivation levels, which enhances external validity. The region did have the highest prescribing rates for ADHD in Scotland [5] during the study period, a potential minor limitation.

Multiple researchers independently analysed the transcripts and then reached consensus, reducing the influence of individual perspectives. Reflexive consideration of positionality further mitigated bias: the status of CS as an undergraduate medical student may have lessened power imbalances during interviews, decreasing the risk of responses being steered toward a particular outcome.

Several limitations warrant consideration. The sample size was small. We mitigated the limited GP sample by using a sampling frame to achieve representation by age, experience, and practice affluence; despite purposive sampling, the distribution of participants by sex was unequal. The more limited psychiatry data primarily served to contextualise and compare with GP findings rather than stand alone for in-depth analysis. Nonetheless, this did not prevent us from meeting the study aims, and the psychiatrists’ input added a useful dimension to the interpretation of GP discussions.

Additional considerations include potential self-selection and nonresponse bias among clinicians who opted to participate, and the possibility of social desirability and recall bias; findings rely on self-reported attitudes and behaviours rather than audited practice. We did not triangulate perceptions with objective indicators (e.g., prescribing records, waiting-time data, or monitoring audits), and member-checking was not undertaken, which may affect confirmability. Although the design was grounded theory-informed, we used the framework method and a prespecified sampling frame; this hybrid approach may have constrained theory generation compared with full theoretical sampling to saturation.

Our sample was limited in composition and did not include patients, CAMHS psychiatrists, locum psychiatrists, or other professional groups, potentially narrowing system-level and lived-experience perspectives. Absence of patient voices in particular may limit conclusions about motivation for self-diagnosis: GP accounts may not fully capture patients’ internal experiences, reasoning, or the socio-contextual factors shaping decisions to seek assessment or treatment.

Finally, while transcripts were double-coded and discussed to consensus, we did not report inter-coder agreement statistics or a detailed audit trail, and conclusions about safety and under-monitoring are based on clinician accounts without chart review or electronic health record verification.

## Conclusions

In this study, GPs perceived increased demand for adult ADHD assessment driven by greater societal awareness. Analysis of GP and psychiatrist responses suggests that social contagion contributes to rising presentations, diagnoses, and prescriptions. Overall, participating GPs viewed this population as underserved. Adults with inattention and impulsivity and those with diagnosed ADHD face limited service availability, with access further constrained by high demand. Primary care clinicians in our sample identified gaps in training, shared-care arrangements, and service accessibility.

Our findings indicate that care for adults with ADHD can be improved through ensuring monitoring of psychotropic medicines, advocating for patients transitioning from childhood to adult services, signposting to education resources, and collaborating with secondary care via further development of shared care agreements. Given the size, urban-rural composition, and ADHD caseload of the health board studied, these conclusions may be indicative of views held by primary care clinicians more broadly.
